# Estimating the quality of eukaryotic genomes recovered from metagenomic analysis with EukCC

**DOI:** 10.1186/s13059-020-02155-4

**Published:** 2020-09-10

**Authors:** Paul Saary, Alex L. Mitchell, Robert D. Finn

**Affiliations:** European Molecular Biology Laboratory, European Bioinformatics Institute (EMBL-EBI), Wellcome Trust Genome Campus, Hinxton, Cambridge, UK

**Keywords:** Metagenomics, Eukaryotes, Genome quality estimation, Metagenome assembled genomes, Malassezia, Bathycoccus

## Abstract

Microbial eukaryotes constitute a significant fraction of biodiversity and have recently gained more attention, but the recovery of high-quality metagenomic assembled eukaryotic genomes is limited by the current availability of tools. To help address this, we have developed EukCC, a tool for estimating the quality of eukaryotic genomes based on the automated dynamic selection of single copy marker gene sets. We demonstrate that our method outperforms current genome quality estimators, particularly for estimating contamination, and have applied EukCC to datasets derived from two different environments to enable the identification of novel eukaryote genomes, including one from the human skin.

## Background

The DNA of microorganisms is routinely extracted, sequenced and assembled into genomes, both from isolate cultures and within the context of metagenomic analyses. Estimating the quality of the recovered genome is crucial, to prevent incomplete or contaminated genomes from being published. Single copy marker genes (SCMGs) are routinely used to estimate the quality of a newly assembled genome. As these genes are expected to occur only once within a genome, comparing the number of SCMGs found within a draft genome to the number of expected marker genes provides an estimation of completeness, while additional copies of a marker gene can be used as an indicator of contamination. This approach has been widely accepted for prokaryotes and eukaryotes alike [1–4]. For prokaryotic genomes, CheckM [4] is the most widely used tool for estimating completeness and contamination, although other approaches have also been used and sets of prokaryotic SCMGs are also provided by BUSCO [2, 5] and anvi’o [6]. CheckM uses an initial set of universal SCMGs to identify the clade of a genome and subsequently uses clade specific sets to estimate the quality.

Similarly, SCMGs have also been used to assess isolate eukaryotic genomes as well. CEGMA [1] used 240 universal single copy marker genes identified from six model organisms to estimate genome completeness. BUSCO superseded CEGMA, with the major advance of curated marker gene sets for several eukaryotic and prokaryotic clades, in addition to the single universal eukaryotic marker gene set. However, while BUSCO (version 3.1) provides sets to estimate completeness of eukaryota, protists, plants and fungi, it remains up to the user to select which is the most suitable set when assessing genome quality. Besides these more universal approaches, others have focused on certain eukaryotic clades: FGMP estimates genome quality of fungal isolate genomes for which it utilises both SCMGs and also highly conserved regions found within fungal genomes [7]. For protists, anvi’o provides a reduced number of profiles from the BUSCO ‘protist’ set to estimate the genome quality of eukaryotic genomes (unpublished).

The past two decades have seen a dramatic advancement in our understanding of the microscopic organisms present in environments (known as microbiomes) such as oceans, soil and host-associated sites, like the human gut. Most of this knowledge has come from the application of modern DNA sequencing techniques to the collective genetic material of the microorganisms, using methods such as metabarcoding (amplification of marker genes) or metagenomics (shotgun sequencing). Based on the analysis of such sequence data, it is thought that up to 99% of all microorganisms are yet to be cultured [8].

To date, the overwhelming number of metabarcoding and metagenomics studies have focused on the bacteria present within a sample. However, viruses and eukaryotes are also important members of ecosystems, both in terms of their numbers and functions [9–12]. Indeed, the unicellular protists and fungi are estimated to account for about 17% of the global microbial biomass. Within the microbial eukaryotic biomass, the genetically diverse unicellular organisms known as protists account for as much as 25% [13].

Despite the increased number of complete and near complete genomes, metabarcoding and metagenomic approaches that have included the analysis of microbial eukaryotes have demonstrated that the true diversity of protists is far greater than that currently reflected in the genomic reference databases (such as RefSeq or ENA). For example, a recent estimate based on metabarcoding sequencing suggests that 150,000 eukaryotic species exist in the oceans alone [14], but only 4551 representative species have an entry in GenBank (15. Nov 2019). Thus, if the functional role of a microbiome is to be completely understood, we need to know what these as yet uncharacterised organisms are and the functional roles that they may be performing.

Currently, one of the best approaches for understanding microbiome function is through the assembly of shotgun reads (usually 200–500 bp long) to obtain longer contigs (typically in the range of 2000–500,000 bp). These contigs provide access to complete proteins, which may then be interpreted within the context of surrounding genes. In the last few years, it has become commonplace to extend this type of analysis to recover putative genomes, termed metagenome assembled genomes (MAGs). MAGs are generated by grouping contigs into sets that are believed to have come from a single organism—a process known as binning [15]. However, even after binning, MAGs vary in their completeness and are typically fragmented, due to a combination of biological (e.g. abundance of microbes), experimental (e.g. depth of sequencing) and technical (e.g. algorithmic) reasons. Furthermore, the computational methods used for binning the contigs can sometimes fail to distinguish between contigs that have come from different organisms, leading to a chimeric genome (termed contamination). This is an issue that has to be taken into account when analysing MAGs [16]. Consequently, genome quality assessment is a crucial part of the process, allowing the identification and selection of high-quality MAGs.

Here, we investigate the performance of current approaches across different eukaryotic clades and describe EukCC, an unsupervised method for the estimation of eukaryotic genome quality in terms of completion and contamination, with a particular view of applying this tool for the assessment of eukaryotic MAG quality.

## Results

### Evaluation of BUSCO across different eukaryotic clades

To determine the applicability of BUSCO 3.1 for evaluating the quality of eukaryotic MAGs, we first tested how the more general eukaryotic BUSCO set performed in terms of assessing the completeness and contamination for a range of eukaryotic isolate genomes. Briefly, fungal and protist genomes were downloaded from the NCBI Reference Sequence Database (RefSeq) and completeness and contamination was estimated using BUSCO in ‘*genome mode*’, which employs AUGUSTUS for gene prediction [17], with the eukaryota SCMG set (‘eukaryota_odb9’). Subsequently, the completeness and contamination of fungi and protist genomes were additionally estimated using the fungal (‘fungi_odb9’) and protist (‘protists_ensembl’) sets respectively. As these genomes are of high quality and manually curated, it was anticipated that they should have very high levels of completeness and minimal levels of contamination.

**Fig. 1:**
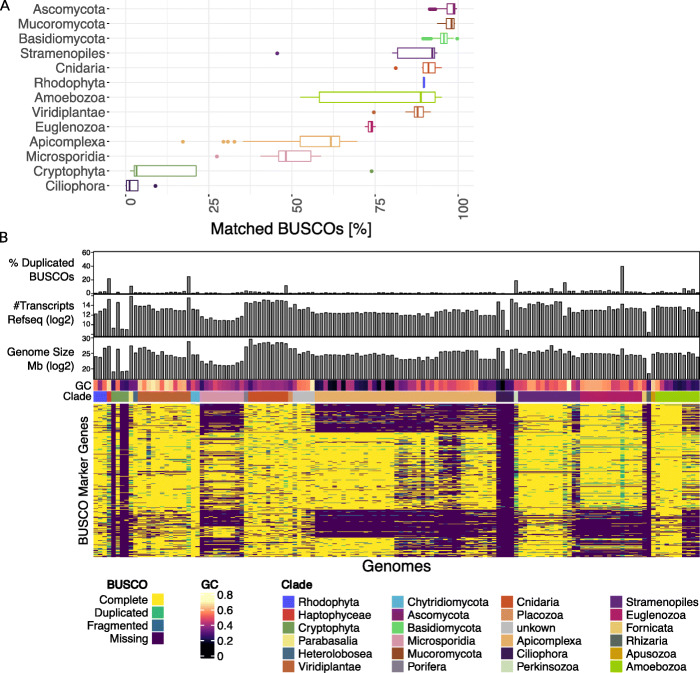
Benchmarking of BUSCO using reference genomes. **a** We downloaded eukaryotic RefSeq genomes excluding bilateria and vascular plants and ran BUSCO in ‘*genome mode*’ using the ‘eukaryota_odb9’ set. For each clade, we summarised the number of BUSCO markers matched. For fungal clades, such as Ascomycota, Mucoromycota and Basidiomycota, most BUSCOs matched a single target—suggesting 100% completeness of the reference genomes. However, in other clades, a substantial fraction of BUSCOs were frequently not matched (Apicomplexa, for example). **b** For species not belonging to fungal clades, we created a matrix using the detailed BUSCO results. Genomes are sorted taxonomically (using the assigned NCBI taxonomy) in columns and the result for each BUSCO in rows. The matrix is coloured according to the BUSCO result, which reports complete, duplicated, fragmented and missing marker genes. Fragmented hits are reported if only part of the BUSCO was detected. Above is shown the percentage of duplicated BUSCOs, the number of the RefSeq transcripts for each genome, the genome size and the GC content. In some clades, there is a clear relationship between the genome taxonomy and missing BUSCOs. In the case of Microsporidia and Apicomplexa, but also for Euglenozoa, this relationship is especially strong

To understand the overlap between the eukaryotic BUSCO set and the selected genomes, we counted the number of matched BUSCOs in each taxonomic clade containing at least 3 reference genomes. While BUSCO reports complete, fragmented and duplicated BUSCOs, for the sake of simplicity we summarised all these as ‘matched’ BUSCOs (Fig. 1a). One of the main applications of BUSCO has been the assessment of fungal genomes, which also represent the most numerous eukaryotic genomes in the reference databases. Thus, it was unsurprising that > 95% of the 303 eukaryotic BUSCOs were matched in genomes coming from Ascomycota, Mucoromycota and Basidiomycota. However, BUSCO performed less well on eukaryotic genomes arising from other taxonomic groups. Notably, the numbers of BUSCOs found in Amoebozoa genomes varied greatly, with a median of 88.78%, but varied between 69.6% for Entamoebidae (number of species, *n* = 4) and 94.9% for the four further Amoebozoa families (*n* = 6). More surprising was that the Ciliophora genomes (*n* = 4) rarely matched BUSCO eukaryotic marker genes, with a median of 1.16% of BUSCOs matched.

We also evaluated the BUSCO protist set in the same way. Somewhat counterintuitively, using this more specific set the mean proportion of matched BUSCOs in Amoebozoa dropped from 88.78 to 78.37%, yet increased for Apicomplexa from 61.72 to 68.37%. In other taxa, such as Stramenopiles, the range of missing BUSCOs increased (Fig. 1S). This suggests that the use of a more specific BUSCO set can improve predictions, but that it does not resolve the problem of inaccurate estimation of completeness in specific clades.

To determine if the underestimation in clades other than fungi is random or caused by systematic biases, we created a matrix containing all found, missing, fragmented or complete BUSCOs in all analysed reference genomes, excluding Basidiomycota, Mucoromycota and Ascomycota (Fig. 1b, see the “Methods” section). We arranged the columns based on the NCBI taxonomy and rows using *k*-modes clustering. Within certain clades, such as Cryptophyta, Microsporidia and Apicomplexa, the same BUSCOs were often missing across a large number of species. For each BUSCO, we evaluated whether it was missing in at least half the species of a given clade. Subsequently, when disregarding any BUSCO missing in at least three clades, the number of BUSCOs in the eukaryota set was reduced from 303 to 86.

Taken together, this shows that the BUSCO eukaryota set does not perform uniformly across all eukaryotic clades. Others have observed similar issues when investigating individual species or clades [18, 19]. We also investigated whether factors, such as genome size, GC content or proteome size, could account for the bias in matching BUSCOs, but taxonomic lineage represented the single strongest signal.

### Influence of gene prediction on BUSCO matches

To understand whether issues with de novo gene prediction could be the cause of the missing BUSCO matches, we additionally ran BUSCO in ‘*protein mode*’ on the genome protein annotations provided by RefSeq and proteins predicted using GeneMark-ES ([20]; Fig. 1S C). When running BUSCO in this mode against RefSeq protein annotations, the number of matched BUSCOs increased overall, indicating that de novo prediction methods do account for some of the loss of sensitivity. However, the general pattern of missing markers across clades remained. Taking Ciliophora as an extreme example, the median of matched markers was 1.2% in ‘*genome mode*’, which was increased to 76.2% using RefSeq annotations. For other clades, the differences were less substantial but still observable. For example, in Apicomplexa 61.7% of BUSCOs were matched using AUGUSTUS, rising to 73.9% using GeneMark-ES and 74.2% with RefSeq annotations. Notably, GeneMark-ES failed to run on several genomes of the Cryptophyta and Ciliophora clades, as well as for the single Rhizaria genome, which BUSCO estimated in ‘*genome mode*’ to have close to 100% missing markers. The primary reason GeneMark-ES did not work for a genome was a lack of suitable training data: out of six failed annotation attempts, five had four or less contigs included in the training phase of GeneMark-ES.

### Establishing specific single copy marker gene libraries

To more accurately compute quality estimates for novel genomes, we wanted to define sets of SCMGs that were comprehensive for microbial eukaryotes, as well as being both sensitive and specific. As shown above, BUSCO produces sets of SCMGs for specific clades which can be more precise in quality estimation. Building on this observation, we aimed at defining multiple sets of SCMGs covering a large range of protists and fungi. We anticipate that a key use case of the marker gene library will be the application of it to poorly characterised genomes, and as such, the genes are likely to be identified by de novo prediction. We therefore chose to (re-)annotate all eukaryotic RefSeq species (not belonging to bilateria or vascular plants) using GeneMark-ES generated gene predictions to more closely represent the use case of metagenomics. In addition, this favours the selection of marker genes which can be predicted using de novo approaches. GeneMark-ES was chosen and applied as we previously demonstrated that the tool worked well across a large range of species and generally performs closer to the RefSeq annotation benchmark. Additionally, we added all eukaryotic species that are used as reference genomes in UniProtKB. The resulting proteins were then annotated with the family-level profile HMMs from PANTHER 14.1 [21] using hmmer (version 3.2) [22]. We choose PANTHER, as amongst tested databases, it has been shown to have the largest coverage of the analysed proteins [23] and because the PANTHER profile HMMs model full-length protein families rather than their constituent globular domains.

In order to increase paralog separation and minimise local matches caused by common domains, we aimed to define profile specific bit score thresholds. To achieve this, we relied on a taxonomically balanced set of species, across which, for each profile, we identified the bit score threshold leading to the highest number of single copy matches (see the “Methods” section).

Thereafter, to define clade specific SCMGs, we first constructed a reference tree for the given genomes using 55 widely occurring SCMGs (from here on termed “reference set”, see the “Methods” section). In each clade of the tree, we checked for SCMGs with a prevalence of at least 98%. A set of marker genes was then defined whenever we found 20 or more PANTHER families in a clade matching the aforementioned prevalence threshold. Using this approach, we were able to define 477 SCMG sets across the entire tree. In contrast to BUSCO and CEGMA, we were not able to identify SCMGs based on the PANTHER models that were applicable to the entire eukaryotic kingdom, but instead found sets applicable to many subclades. While this is desirable for specificity, the obvious drawback is knowing which set is the most appropriate to use—it would be impractical to manually assign the most appropriate set (especially if a large number of different genomes were to be assessed). Thus, we developed EukCC, a software package to select the most appropriate SCMGs, and use these to estimate genome quality.

### Automatically selecting the appropriate single copy marker gene set

To select the most specific set of SCMGs for a novel genome of unknown taxonomic lineage, EukCC performs an initial taxonomic classification by annotating the de novo predicted proteins using the 55 widely occurring SCMGs reference set. Pplacer [24] is then applied to phylogenetically contextualise each match within the reference tree. Tracing each placement in the tree, EukCC determines the lowest common ancestor (LCA) node for which an SCMG set is defined in the database.

As may be expected, while pplacer often places all sequences in a simple, narrow region of the reference tree, occasional placements occur within inconsistent, distantly related clades. In such cases, no single set of SCMGs may encompass all locations. To overcome these cases, the SCMG set that encapsulates the largest fraction of the placements is located. While this process overcomes cases where outlying placements occur due to incorrect or inconsistent placements, this approach may select an incorrect SCMG set if the matches to the reference SCMGs from a novel genome cannot reliably be placed in the tree. To help control for this, EukCC always reports how many profiles are covered in a set and provides the option of plotting the placement locations (Fig. 5S). Thus, in a situation where a set was chosen that only encompasses a fraction of the reference SCMGs, a more in-depth analysis of this MAG could, and should, be carried out.

After the initial placement, EukCC assesses the completeness and contamination in a second step by annotating all proteins with the profiles that are expected to be single copy within the assigned clade. EukCC then reports the fraction of single copy markers found and the fraction of duplicated marker genes, corresponding to the completeness and contamination score, as provided for prokaryotes by CheckM. Additionally, EukCC uses the inferred placement to give a simple phylogenetic lineage estimation based on the consensus NCBI taxonomy of the species used to construct the chosen evaluation set.

### Comparison of BUSCO to EukCC quality estimates

Having established new sets of SGMGs and having developed EukCC for their selection, we next evaluated the accuracy of our approach for estimating completeness and contamination. To do so, we used both EukCC and BUSCO to estimate the completeness and contamination of 19 RefSeq genomes, from 6 different clades, that were not used to establish the EukCC SCMGs. As these were complete genomes, we simulated varying amounts of completeness and contamination (see the “Methods” section). During the preparation of this manuscript, an updated version of BUSCO was produced, version 4, which introduced a similar concept as described above for EukCC for automatically selecting gene marker datasets. For the sake of completeness, we have also included BUSCO 4.0. For BUSCO 3.1, we used the taxonomy assigned to each genome to select the most specific BUSCO sets, EukCC and BUSCO 4.0 dynamically selected the SCMGs set from their respective library of clade specific SCMGs. As we showed earlier that the de novo gene prediction can have an influence on the BUSCO results, we ran BUSCO using AUGUSTUS as well as in ‘*protein mode*’ on GeneMark-ES predicted proteins, which are also used by EukCC. It is worth noting that due to the limited availability of RefSeq genomes, the genomes selected in this benchmark were partially (10 out of 19) already included in OrthoDB v10v1 (odb10) and as such confound the benchmark in favour of BUSCO 4.0. This is particularly true for the Stramenopile and the Viridiplantae clade, from which all of the benchmark genomes are included in odb10.

When estimating completeness across simulated genomes with no added contamination, EukCC performed better than BUSCO 3.1 or 4 using AUGUSTUS and better than BUSCO 3.1 with GeneMark-ES. Completeness estimates between BUSCO 4.0 and EukCC, both using GeneMark-ES, were within 2.5% for genomes of at least 80% completeness. With increasing contamination, EukCC’s estimates show a lower deviation from the ground truth. As BUSCO 4 overall outperforms BUSCO 3 in our analysis, we will focus on this version from here on when referring to BUSCO.

**Fig. 2:**
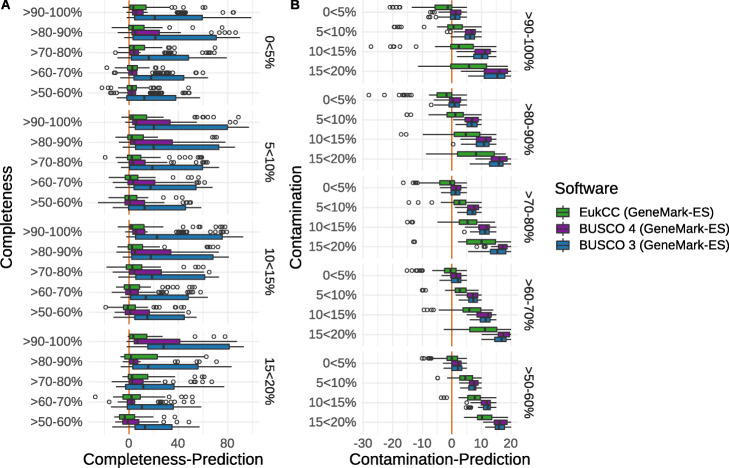
Comparison of EukCC and BUSCO using simulated data. We compared EukCC to BUSCO (versions 3.1 and 4.0) using a set of 19 genomes from RefSeq belonging to Alveolates, Amoebozoa, Apusozoa, Fungi, Rhizaria, Stramenopiles and Viridiplantae. We fragmented the genomes and added varying amounts of contamination from another genome in the same clade. We then ran BUSCO and EukCC to estimate completeness and contamination. The red line highlights 0% deviation from the ground truth. **a** We defined completeness in BUSCO as 100% minus missing BUSCOs. For genomes with a contamination between 0 and 5%, EukCC underestimated completeness with a median of 2.74%, while BUSCO 3.1 underestimates the completeness across all genomes with a median above 20%. BUSCO 4.0 underestimates completeness on average by 5.75%. With increasing amounts of contamination, EukCC underestimates more rarely. Only when genome completeness falls below 50% and/or contamination exceeds 15% does EukCC consistently overestimate completeness. **b** To evaluate contamination we counted the number of duplicated BUSCOs or marker genes (in the case of EukCC). For genomes with 0–5% contamination and high completeness (> 90%), EukCC overestimates contamination, but by below 5%. With increasing amounts of contamination, EukCC tends to underestimate contamination, but outperforms BUSCO 4.0, which consistently underestimates contamination by a larger fraction

BUSCOs completeness estimates for simulated genomes with more than 95% completeness and no contamination, using AUGUSTUS or GeneMark-ES, had a mean deviation from the ground truth of 5.75% with GeneMark-ES having the lower standard deviation of 21%. Meanwhile, EukCC’s estimates are more accurate with a deviation of 2.74% and a standard deviation of 13% (Fig. 2a). Within clades, there is variation to be noted between BUSCO and EukCC: using this benchmark BUSCOs completeness estimate accuracy varies amongst clades. Out of all the tested clades, BUSCO performed best for Fungi, Alveolates and Stramenopiles, but underestimates completeness in Amoebozoans. EukCC’s estimates are comparable for Fungi and Alveolates, but more reliable for Amoebozoans. BUSCO outperforms EukCC in the Stramenopile clade, where EukCC has a larger standard deviation and mean, but notably all genomes of this clade benchmark are included in odb10 and so the benchmark is not independent from the training data for BUSCO, where it is independent for EukCC. Between both tools, it is notable that clades with little training data, such as Stramenopiles and Amoebozoa, are more often underestimated in their completeness.

To demonstrate EukCC’s performance in estimating contamination, we also assessed the contamination estimates against the known value of contamination, against a background of increasing levels of genome completeness (Fig. 2b, Additional file 1: Fig. S2). Thus, contamination from other genomes belonging to the same clade was added to complete or partially fragmented genomes (see the “Methods” section). We investigated in-clade contamination first, as opposed to out of clade contamination, as this form of contamination is more likely given current binning algorithms. For almost complete genomes, those with > 90% completeness and simulated contamination < 5%, EukCC deviates from the expected contamination estimate on average by 2% for Fungi and Alveolates. At lower levels of completeness (60–80%), EukCC’s contamination estimates are less accurate, but as completeness increases (> 90%), the accuracy of contamination estimation increases, with a median error of < 2% for genomes with contamination below 10%. Overall, as genomes include increasing amounts of contamination, EukCC begins to overestimate completeness, e.g. by ~ 5% for fungal genomes with expected completeness 60–70% and a contamination of 10–15% (Additional file 1: Fig. S2). This is somewhat to be expected, as there is a greater chance of finding an expected marker gene in the contaminating contigs, leading to inflated completeness. While EukCC tends to overestimate contamination in the range of 0–5% contamination, BUSCO tends to underestimate contamination across all simulated genomes containing more than 5% contamination.

Next, we ran the same benchmark with contamination added from genomes outside the clade, to understand if EukCC’s estimates remain stable. Overall, we saw a slight decrease in the accuracy of the contamination estimate, which resulted from a wider variation in the contamination estimates. However, completeness estimated remained consistent with the previous within clade contamination, with genomes overestimated on average by 1.6% for genomes with a simulated completeness > 90% and contamination 5–15% (Additional file 1: Fig. S3). To understand the cause of the slight overestimation of genome completeness with increasing amounts of added contamination using EukCC, we added contamination in the form of random DNA to the genomes and repeated the genome completeness and contamination assessments. In this case, the contamination estimate was not affected by the added contamination, confirming the hypothesis that overestimation of completeness is caused by contaminating contigs by chance providing the correct, yet missing, SCMGs.

Another advantage of EukCCs for estimating completeness and contamination of MAGs is the uniform distribution of the SCMGs across the entire genome. To demonstrate this, we randomly sampled 5 kb fragments and computed the Pearson correlation between the sampled size and the recovered marker genes for all species used within this benchmark. All sets used in this test showed linearity with a Pearson correlation coefficient of at least 0.95, indicating a uniform distribution of the marker genes across the genome. Nevertheless, it remains possible that shorter contigs that are assigned to a MAG may lack any marker gene, so are not evaluated as part of the genome quality estimates performed by EukCC. To highlight this, EukCC outputs the fraction of genome that cannot be assessed due to this feature.

Across all simulated genomes, EukCC could estimate genome quality starting from a completeness of around 50%. Genomes less complete than this were often not able to be processed using the self-training mode of GeneMark-ES. To overcome these shortcomings of GeneMark-ES, we developed a python package called *pygmes* which allows the use of pre-trained GeneMark-ES models for over 700 species. Briefly, *pygmes* tries to run GeneMark-ES in self-training mode first; if this fails, it will predict proteins using GeneMark-ES with five pre-trained GeneMark-ES models from different taxonomic clades. Using the best proteome (in terms of number of amino acids/proteins), it will predict the taxonomic lineage against a UniRef50 database with diamond. Using this taxonomic assignment, the model with the largest taxonomic overlap amongst the 700 available GeneMark-ES training models will be chosen and applied to predict the final proteome. Users can opt to rely on this library of models by providing the ‘--pygmes’ flag to EukCC.

Overall, in this benchmark, we found that BUSCO 3.1 and 4 tend to underestimate contamination in genomes of high completeness (Additional file 1: Fig. S2). BUSCO 4.0 performs better than BUSCO 3.1 and provides more reliable completeness estimates when paired with GeneMark-ES instead of the default AUGUSTUS gene caller. Meanwhile, EukCC tends to underestimate completeness and overestimates contamination (albeit at low rates), which leads to more conservative, yet more accurate genome quality estimates.

### Comparing gene predictions from GeneMark-ES to RefSeq

As BUSCO’s performance increased when using GeneMark-ES compared to AUGUSTUS, we investigated how well the GeneMark-ES predicted proteins overlap with annotations from RefSeq. For a taxonomically balanced subset of 89 eukaryotic genomes, we predicted proteins de novo using GeneMark-ES and cross referenced SCMGs used by EukCC against RefSeq annotated sequences from the same species using DIAMOND ([23]; see the “Methods” section). We then generated a pairwise alignment between the predicted (query) protein and the best hit from the reference set and counted the gaps (irrespective of length) in both the reference and the query. Pairwise alignments with few gaps generally involve proteins of the same length. In the relatively few cases where there were a larger number of gaps (> 10 gaps), these were introduced because the GeneMark-ES proteins were smaller compared to RefSeq, suggesting that GeneMark-ES does miss a small subset of exons. Despite this, the assigned RefSeq proteins and the corresponding GeneMark-ES proteins were found to have a generally similar length distribution. Together, this suggests that the SCMGs chosen by EukCC and predicted by GeneMark-ES are similar to the annotations in RefSeq (Additional file 1: Fig. S5).

### Application of EukCC for the evaluation of MAG quality

Having established the utility of EukCC on the simulated benchmark, we applied it to metagenomic datasets. As a first example, we investigated samples from the skin microbiome, a relatively well characterised microbiome, where the community has low diversity and is known to include many fungal species, many of which have been isolated and their genomes sequenced [25, 26]. These features provided the best chance of producing de novo assembled eukaryotic MAGs for which we could estimate the quality using EukCC and independently verify their quality using reference genomes. Furthermore, given that BUSCO performs well for fungal genomes, this would provide additional validation of the EukCC results.

We retrieved the sequencing data for the largest publicly available human skin microbiome study (accession PRJNA46333 [27, 28]), which comprises 4009 individual shotgun sequencing runs, from which 2488 runs can be assigned to 15 individuals. Following successful assembly of 3910 runs with metaSPAdes [29] and binning with CONCOCT ([30]; see the “Methods” section), 2497 of the assembled runs produced bins, generating 56,610 bins in total. As these bins were expected to be a mixture of bacterial and eukaryotic genomes [31, 32], a top level classification was performed of all bins using EukRep [33] to identify any bin containing at least 1 Mb of predicted eukaryotic DNA, reducing the number of bins from 56,610 to 434 (with the bins hereafter referred to as a MAG).

Using EukCC, we could predict the MAG quality for 233 out of the 434 MAGs. We then assigned reference genomes to as many MAGs as possible, by finding the closest GenBank entry for each based on Mash distances ([34]; see the “Methods” section). 93.13% of the MAGs (217 out of 233) could be assigned to a fungal reference genome with a Mash distance < 0.1, corresponding to average nucleotide identity (ANI) of ~ 90% or above. To verify the EukCC genome completeness estimates, we compared the alignment fraction of the reference to the predicted completeness of EukCC for all MAGs. For those MAGs that could be aligned to a reference genome with an ANI > 95% and had a predicted contamination below 5% and a completeness > 50%, the median difference between alignment fraction and predicted completeness was 4.6%, with EukCC underestimating the completeness (Additional file 1: Fig. S4 B). BUSCO 4.0 underestimated completeness in the same MAGs by 9.8% while FGMP [7] (another fungal genome quality estimator) overestimated completeness by 11.24% (Additional file 1: Fig. S4 D).

Next, we removed the redundancy between the MAGs (based on the assignment to the same reference genomes and retaining the most complete MAG, with a contamination < 5%). This yielded a non-redundant set of 5 MAGs, corresponding to *Malassezia restricta* (with a EukCC reported completeness of 92.84% and a contamination of 1.38%), *M*. *globosa* (completeness 84.87 and contamination 1.49%), *M*. *sympodialis* (completeness 85.56% and contamination 0.79), the unclassified *M*. *sp*. (completeness 83.05% and contamination 2.23%) and *M*. *slooffiae* (completeness 81.21% and contamination 2.37%). The average nucleotide identity (ANI) to the respective reference genome was above 98% for all MAGs but *M*. *sp*. (ANI 93.7%).

We found five additional MAGs that we could not assign to any known *Malassezia* species, but were identified by EukCC as likely to belong to the *Malassezia* genus. We computed the mash distance between all MAGs and determined that they belong to the same unknown species. After dereplication, the representative MAG, from here on termed ‘Novel_Malassezia_MAG’, was estimated to have a completeness of 93.43% and a contamination of 1.84%.

**Fig. 3:**
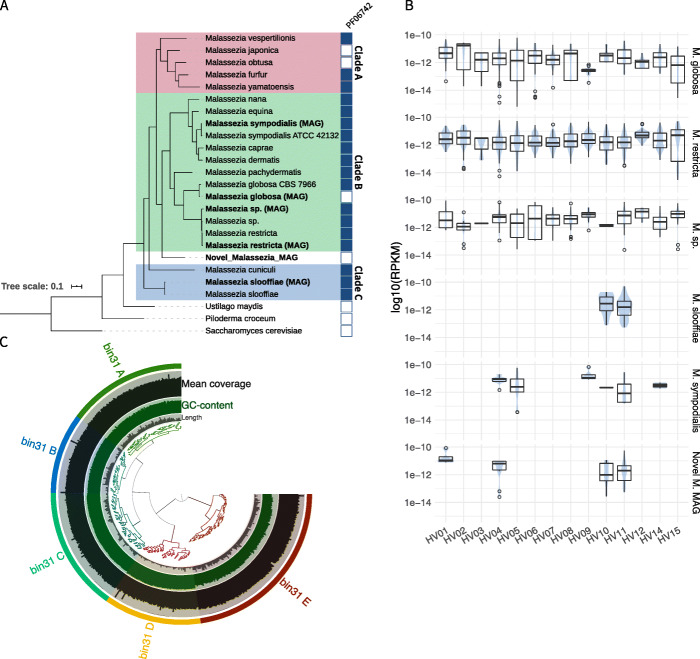
Recovery of MAGs belonging to *Malassezia*. We assembled 3910 metagenomes and could recover almost complete MAGs of *M*. *globosa*, *M*. *restricta*, *M*. *sp*., *M*. *sloofiae* and *M*. *sympholidalis*. Additionally, we recovered a *Malassezia* MAG with no known matching species. **a** Using four genes occurring in single copy in all representative *Malassezia* species, in the recovered MAGs as well as in *S*. *cerevisiae* and two species of *Basidiomycota*, we constructed a phylogenetic tree with MAFFT and FastTree2. The tree recapitulates the clustering suggested by Wu et al. [26], consisting of three clusters A, B and C. All recovered MAGs cluster next or close to their assigned species (bold). The MAG representing the unknown species (‘Novel_Malassezia_MAG’) is clustered within the *Malassezia* clade, confirming the previous annotation. **b** For each MAG, we counted the Reads Per Kilobase of transcript per Million mapped reads (RPKM) if more than 30% of the genome was present in a sample. Using this approach, we could detect *M*. *globosa*, *M*. *restricta* and *M*. *sp*. across all individual subjects. The less prevalent *M*. *sloofiae* and *M*. *sympholidalis* could only be found in 2 and 6 individuals, respectively. The ‘Novel_Malassezia_MAG’ could be found in four subjects. **c** We analysed the MAG using anvi’o’s *refine* method. All clusters created by anvi’o based on *k*-mer frequency and coverage have similar coverage and could be annotated as *Malassezia* using UniRef90. Cluster E has a lower GC content but comparable coverage to other clusters

Wu et al. [26] reported that *Malassezia*, in contrast to other Basidiomycetes, should contain the gene family that matches the Pfam entry DUF1214 (Pfam accession PF06742). We could verify the presence of this gene family in all reference *Malassezia* genomes except *M*. *japonica* and *M*. *obtusa*. We could also find this gene family in the MAGs assigned to *M*. *restricta*, *M*. *sloofia*, *M*. *Sp*. and *M*. *sympodialis*, but not in the ‘Novel_Malassezia_MAG’ nor in the MAG assigned to *M*. *globosa*. As both MAGs are predicted to be incomplete, this protein family could be missing by chance or due to misclassification of the MAGs. To further characterise the novel and the recovered Malassezia MAGs, we identified four SCMGs present in all *Malassezia* as well as in *Saccharomyces cerevisiae*, *Piloderma croceum* and *Ustilago maydis* (see the “Methods” section). We used members of these protein families to build a tree that included all recovered non-redundant MAGs and all representative genomes from the *Malassezia* clade, as well as the aforementioned fungi. In the resulting tree, rooted using *S*. *cerevisiae* as an outgroup, all MAGs cluster next to or close to their assigned reference genome. The tree recapitulates the three cluster structure first described by Wu et al. [26]. The ‘Novel_Malassezia_MAG’ representing an unknown species is located within the *Malassezia* clade, and might be a member of clade B, confirming the taxonomic assignment by Mash and EukCC (Fig. 3a).

To investigate the prevalence of the five recovered MAGs, we aligned the reads from 1488 skin metagenomes belonging to 15 individuals to the MAGs and computed the Reads Per Kilobase of transcript per Million mapped reads (RPKM) of unique reads for samples if 30% of the target MAG was covered. Using this approach, we identified *M*. *globosa*, *M*. *sp*. and *M*. *restricta* in all individuals of this study (*n* = 15). The novel *Malassezia* species was present in 4 different individuals, which was more prevalent than *M*. *sloofia* (*n* = 2) and close to the prevalence of *M*. *sympodialis* (*n* = 6) (Fig. 3b).

We then inspected the potentially novel *Malassezia* species genome using anvi’o *refine* [6] and identified five contig clusters (Fig. 3c). Each subcluster was taxonomically analysed using matches to Uniref90 and could be associated to the genus *Malassezia* with a majority vote of at least 60% of the sampled proteins (see the “Methods” section). We also looked at the density of marker genes in each subcluster. Clusters A, B, C and D contribute 24.7%, 15.6%, 19.3% and 7.3% respectively and have a similar marker gene density between 13.7 and 16.5%/Mb. Cluster E contributes 28.0% completeness to the MAG overall, but has a lower marker gene density of only 9.2%/Mb (Additional file 5: Table S4). While Cluster E contains more contigs with lower GC than the other groups, the inclusion in this MAG is supported by the even coverage as well as the taxonomic assignment to the *Malassezia* genus.

### Applying EukCC to a *Bathycoccus* MAG from TARA Ocean data

Having established that EukCC quality estimates were accurate in a well characterised community, we then tested it on samples in which we expect a more diverse range of eukaryotes, beyond fungi. To do so, we focused on the eukaryotic enriched samples (size fractionated samples in the range 0.5 μm to 2 mm (protists size fraction, study: PRJEB4352)) from the TARA Oceans project [10]. As a prelude to investigating eukaryotes from this biome, we randomly selected 10 out of the 912 available runs.

After assembly and binning of the sequences belonging to the run ERR1726523 (see the “Methods” section), we identified a 13 Mb bin that EukCC estimated to have a completeness of 87.62% with a contamination of 0.32%. EukCC inferred a taxonomic placement in the order *Mamiellales* (green algae). Using Mash, we compared this MAG, from here on out termed ‘TARA_1’, to eukaryotic genomes in GenBank, as well as to MAGs published by Delmont et al. [33], which were obtained by assembling and binning data from the prokaryotic size fraction of the TARA Ocean project. The best match within GenBank is the entry *Bathycoccus sp*. *TOSAG39-1* (GCA_900128745.1, 10 Mb), with a Mash distance of 0.041. The taxonomy of the GenBank entry confirmed the EukCC inferred lineage and chosen SCMG set. In a pairwise alignment between ‘TARA_1’ and *TOSAG39-1* using dnadiff, 52.01% of the MAG covered 78.97% of the reference genome with an ANI of 96.08. The identified reference genome was published by Vannier et al. [35] by merging four single-cell amplified genomes (SAGs). Vannier et al. estimated their SAG to be 64% complete using eukaryotic core genes from CEGMA. We estimated the quality of the SAG using BUSCO 4.0 and EukCC, which assessed the completeness to be 56.2% and 59.65% with a contamination of 7.4% and 14.04% respectively (See Table 1). The considerable amount of contamination may have resulted from the merging of the SAGs. The closest match of ‘TARA_1’ to the published TARA MAGs was the entry ‘TARA_PSE_MAG_00140’ with a Mash distance of 0.006. When comparing ‘TARA_1’ to the ‘TARA_PSE_MAG_00140’ MAG using a pairwise genomic alignment, 89.8% of ‘TARA_1’ covers 94.0% of ‘TARA_PSE_MAG_00140’ with an ANI of 99.49%.

**Table 1:** Genome quality metrics for the three genomes. BUSCO 4.0 and EukCC produce similar estimates for all three genomes. The MAG produced by Delmont et al. is substantially more complete than the next best entry in GenBank. The ‘TARA_1’ MAG is more complete and more continuous than both

	EukCC		BUSCO 4.0			
Genome	Compl. (%)	Cont. (%)	Compl. (%)	Cont. (%)	Size [Mb]*	N50 [Kb]*
*TOSAG39-1*	59.65	14.04	56.2	7.4	10.0	14.1
TARA_PSE_MAG_00140	82.46	0.0	82.2	0.1	12.7	8.8
TARA_1	87.62	0.32	88.8	0.3	13.1	18.5

*Scaffold size/N50

To check for assembly and binning errors, we again analysed the MAG using the bin refinement method in anvi’o (Additional file 1: Fig. S6): the anvi’o clustering divides the bin into two main clusters. Both clusters share similar GC content and coverage. From each cluster, we inferred the taxonomic annotation by comparing a subsample of up to 200 proteins against Uniref90. For all analysed clusters, the consensus lineage ended at the genus *Bathycoccaceae*, indicating a consistent MAG with no significant contamination.

In summary, the genome quality estimates of our TARA_1 MAG compares favourably to both the GenBank entry *TOSAG39-1* or the MAG TARA_PSE_MAG_00140, in terms of completeness and continuity. This example highlights that unsupervised recovery of eukaryotic MAGs at scale is feasible, with quality estimates on *a par* with carefully curated MAGs.

## Discussion

Microbial eukaryotes represent a largely unexplored area of biodiversity. The use of modern genomic and metagenomic approaches are beginning to provide access to the genetic composition of these hitherto unknown organisms. However, in this study, we have demonstrated that widely used tools for estimating eukaryotic genome quality (completeness and contamination) do not work uniformly across all microbial eukaryotes, which limits their application—for example within large scale metagenomic pipelines.

Our results also highlight that the quality of the gene prediction step influences the quality estimates given by BUSCO—using NCBI RefSeq annotations instead of AUGUSTUS gene predictions raised the predicted average quality of the tested genomes. We could show that BUSCO 3.1 was not suitable to be used in a metagenomic context, as the eukaryota SCMG set consistently underestimated genome completeness within certain clades. This within-clade error cannot be explained by low quality reference genomes, but rather is indicative of a suboptimal eukaryota set. This issue has been overcome by BUSCO 4.0, which now offers an automated approach for marker gene set selection, similar to what has been proposed for EukCC. We showed that while BUSCO 4.0 has major advantages over BUSCO 3.1, BUSCO 4.0 still underestimates contamination across multiple clades.

To overcome many of these limitations, we have developed EukCC, a novel tool to estimate microbial eukaryotic genome quality. EukCC uses a reference database to dynamically select the most appropriate out of 477 single copy marker genes sets. This set is then used to report genome completeness and contamination, as well as a taxonomic placement. Comparing EukCC and BUSCO 4.0 proved to be difficult due to the limited availability of novel microbial RefSeq genomes, which have not been included in neither EukCC’s training set nor in OrthoDB v10. Even though the chosen benchmark was not independent for BUSCO 4.0 we showed, using simulated data, that EukCC estimates genome quality across several taxonomic clades and performs on a par with, or better than, BUSCO. In addition, EukCC provides a more conservative contamination estimate across all tested clades, which is crucial to the field of metagenomics, where genomes are more likely to contain contaminating DNA. EukCC works independently of user input and can thus be used to analyse potential eukaryotic genomes from unknown species in an unsupervised environment. We showed that EukCC can identify contamination from similar and more distantly related species within a MAG. Nevertheless, it is worth noting that removal of contamination from foreign species is a topic that has recently been addressed by others, with software solutions such as CAT [36]. We think that removal of contigs from distantly related species, say a fungal contig in a Chlorophyta MAG, will be possible based on such approaches and that additional verification steps should always be considered when trying to assess the quality of MAGs.

Nevertheless, there is a clear connection between the number of known species in a taxonomic group and the performance of EukCC. Some eukaryotic clades have very few high-quality reference genomes. For example, at the time of writing, Apusozoa, Rhizaria and Cryptophyta as well as Rhodophyta each have less than 10 reference genomes. While the current version of EukCC is known to perform better for more deeply sampled clades, we have demonstrated that the general framework can deliver consistent and high-quality estimates across a broad taxonomic range. Thus, we aim to update the database regularly in order to build on growing public data and improve our performance across all clades.

Using EukCC, we are now able to systematically screen large libraries of previously ignored or unanalysed bins from published shotgun metagenomes. We have demonstrated that by reanalysing published skin metagenomes, we could find a novel species prevalent in 4 of the sampled individuals. This novel species belongs to the well sampled *Malassezia* genus and could prove interesting in the context of understanding the skin microbiome. We have additionally demonstrated that current metagenomic techniques are also able to recover near complete eukaryotic genomes from more complex biomes, such as marine environments. More importantly, we showed that the combination of tools such as EukRep and EukCC can achieve comparable levels of genome quality compared with more manual approaches (as was performed for TARA_PSE_MAG_00140), by offering a scalable approach for the generation of eukaryotic genomes from metagenomic datasets.

## Conclusion

With EukCC, we present an easy to use tool to estimate genome quality metrics for microbial eukaryotes and have demonstrated a substantial improvement in the applicability of EukCC compared to other tools. While this tool was developed with application to MAGs in mind, we do not see any limitation within EukCC to prevent it from being applied to SAGs, or even isolate genomes. To demonstrate the applicability of EukCC, we have identified two novel eukaryotic genomes from metagenomic samples and have subsequently verified the quality of these genomes using a variety of approaches. EukCC provides the first step of many to assess the quality of eukaryotic MAGs and offers a way to select those that are likely to represent high-quality genomes.

## Methods

### Evaluation of BUSCO results

To evaluate BUSCO 3.1.0, we downloaded genomes and corresponding annotations for 418 eukaryotic reference species from RefSeq (September 26, 2019), excluding those belonging to bilateria or vascular plants. Each genome was annotated using GeneMark-ES (version 4.38, parameters: ‘-v -fungus -ES -cores 8 -mincontig 5000 -sequence input.fa’). We then ran BUSCO (version 3.1) in using the ‘eukaryota_odb9’ BUSCO set in ‘*genome mode*’ using AUGUSTUS (version 3.3.2) as well as in ‘*protein mode*’ for both the RefSeq annotated proteomes as well as the GeneMark-ES predicted proteins. This procedure was then repeated for the ‘protist_ensemble’ BUSCO set. To compare the BUSCO results to EukCC, we defined completeness as 100 minus the fraction of missing BUSCOs and contamination as the fraction of duplicated BUSCOs.

For Fig. 1b, all reported BUSCOs in all analysed genomes were displayed using ComplexHeatmap [37] in R 3.5.1 [38] and clustered the rows using ‘klaR’ [39].

### GeneMark-ES de novo protein prediction comparison

Following this, we compared RefSeq provided annotation and proteins predicted from GeneMark-ES (parameters: ‘-v -fungus -ES -cores 8 -mincontig 5000 -sequence input.fa’). For each genome, we ran the BLASTp option from DIAMOND [40] on the proteins used by EukCC to estimate genome quality matching against the RefSeq annotated proteome of the same species. For the best hit, we aligned both sequences using MAFFT [41]. Subsequently, we compared the length distributions between GeneMark-ES and RefSeq annotated sequences. Additionally, we counted the number of gaps within the alignment occurring in either the reference or the query sequence. Analyses were performed using R 3.5.1 and plots were generated using ggplot [42].

### EukCC reference database creation

To build EukCC’s database, we downloaded the genomes of 754 eukaryotic species from NCBI GenBank and RefSeq, all of which were either marked as representative genomes (August 1, 2019) or used as UniProt reference proteomes (May 28, 2019) [43] excluding those belonging to bilateria or vascular plants (Additional file 2: Table S1). Following this, we predicted the proteome of each genome using GeneMark-ES and annotated the resulting proteins using PANTHER families 14.1 with hmmer 3.2.1 (Additional file 1: Fig. S7 A)). During this process, 20 genomes were excluded due to GeneMark-ES failing to produce an output, reducing the number of species to 734. The failure was mostly caused by fragmented reference genomes, making it impossible for GeneMark-ES to pass the training step.

Subsequently, using the annotated proteins of a taxonomically balanced subset of species, we defined bit score gathering thresholds for each PANTHER profile HMM. For this, we chose at most 30 genomes per major sub-clade of eukaryota (e.g. Opisthokonta, Amoebozoa, Alveolata) and sampled evenly across all phyla below. Within these species, we identified the bit score value maximising the number of single hits for each profile HMM.

After applying the bit score thresholds across all annotated species, we searched for profiles covering all species as single copy markers. As no single copy markers spanning all species could be found, we used a greedy algorithm to define a reference set of overlapping single copy marker genes. The resulting reference set contained 55 profiles, covering each species within the training set as a single copy marker between 3 and 34 times. The single copy proteins belonging to each profile HMM within the reference set were aligned using MAFFT and horizontally concatenated. Consequently, we used this alignment to build a reference tree using FastTree2 with default settings [44].

Following this, we identified 477 sets of single copy genes with a single copy prevalence cut-off of 98 in each clade of at least 3 species.

### Overview of the EukCC algorithm

As a first step, EukCC uses Genemark-ES to predict proteins in the input genome (Additional file 1: Fig. S7 B). The EukCC pipeline then performs a two stage analysis to determine the best set of SCMGs for downstream analysis. The first stage uses the reference set to define a first approximate taxonomic classification of the MAG to enable the placement in the precomputed reference tree using pplacer version v1.1.alpha19 [24]. For each protein, the best placement as indicated by the posterior likelihood is chosen. Using these placements, EukCC relies on ete3 to compute the lowest common ancestor (LCA) or the highest possible ancestor (HPA) for which a set of single marker genes exist [45]. In a second stage, the HMMs defined in the chosen SCMG set are scanned against the predicted proteome using hmmer. The fraction of existing profiles is reported as completeness, and the fraction of duplicated markers is reported as contamination. Finally, EukCC reports a lowest common ancestor lineage of the input genome, based on the species within the marker set.

### Evaluation data creation

In order to benchmark EukCC and BUSCO with known data, we created in silico fragmented and contaminated genomes. For this we chose RefSeq genomes across all relevant taxonomic clades, which were not included in the initial training data. From each clade, we selected up to 4 species to evaluate completeness and contamination. If a selection of species could be made, we first included species from a rank not included in the training set, prioritising novel phyla over novel order and so forth. As GeneMark-ES failed to predict proteins for all considered Cryptophyta species the clade was omitted from the benchmark. We then created fragments by stepping along chromosomes with step size chosen from a Poisson distribution (rpois(*n*, *λ* = 100) and a minimum step size of 2000. Fragments were rejected or included at random to create a genome of a target size fraction. Contaminating contigs were sampled from different species from the same clade and were fragmented in the same way and combined to make a test genome (Additional file 3: Table S2). Additionally, we simulated contamination by sampling from genomes of a different clade (Additional file 4: Table S3).

### Benchmark and comparison of EukCC to BUSCO

Following the creation of the benchmark data, we ran BUSCO (version 3.1) in ‘*genome mode*’ using the AUGUSTUS gene predictor (version 3.3.2) on the simulated genomes. For each genome we used the most suitable set of BUSCOs for the data. For example, when assessing a protist genome, we used the ‘protists_ensembl’ set, and for fungi, we used the universal ‘fungi_odb9’ set. Notably, we used the protist set to evaluate the Alveolata species, as BUSCO’s performance decreased when using the more specific ‘alveolata_stramenophiles_ensembl’ set. We then used the ‘shortsummary*’ files from which we extracted the percentage of missing and duplicated marker genes. We defined completeness as 100 minus the percentage of missing BUSCOs, thus also including fragmented BUSCOs in the completeness score. Additionally, we ran BUSCO in ‘*protein mode*’ using proteins predicted by GeneMark-ES (parameters: ‘-v -fungus -ES -cores 8 -mincontig 5000 -sequence input.fa’). BUSCO 4.0 (version 4.0.5) was run with the flag ‘--auto-lineage-euk’ and allowed to automatically assign the correct marker gene set. EukCC was used with default parameters and database version 1. We discarded any simulated MAG from the benchmark for which not all three methods could produce a quality estimate. Finally, we obtained 678 results per evaluated algorithm, which were aggregated with R using dplyr and plotted using ggplot.

### Assembly and binning of skin metagenomic datasets

We downloaded 3963 shotgun metagenomic datasets from the skin metagenome study PRJNA46333. Each dataset was assembled using metaSPAdes (version 3.12, default parameters in metagenomics mode [-meta]) and binned using CONCOCT (version 1.0) as part of the metaWRAP (version 1.1) [46]. Subsequently, we estimated the genomic composition in each bin using EukRep (version 0.6.5) and bins with more than 1 Mb eukaryotic DNA were selected for further analysis. Selected ‘eukaryotic’ bins were then analysed using EukCC and compared to RefSeq and GenBank (both retrieved September 26, 2019) entries by comparing Mash distances (version 2.2.2 default parameters) and subsequently using dnadiff (from the mummer package, version 3.23) for the top hit, if the Mash distance was below 0.1 [47].

### Tree building and analysis of skin MAGs

To construct a phylogenetic tree for the 6 selected skin MAGs, 19 reference genomes of 16 *Malassezia* species and *Saccharomyces cerevisiae*, *Ustilago maydis* and the GenBank entry of *Piloderma croceum*, we identified 4 SCMGs genes used by EukCC found in all genomes: PTHR10383, PTHR11377, PTHR12555 and PTHR15680. Using MAFFT, in einsi mode, we aligned the protein sequences for each PANTHER entry before building a concatenated alignment file, which was used as input to FastTree2 to build the tree, using default settings. We visualised and rooted the tree using *S*. *cerevisiae* as an outgroup with iTOL v5 [48]. Using hmmer 3.2.1 (hmmscan -cutga) we searched for the Pfam [49] entry DUF1214 (Pfam accession PF06742, Pfam version 32) in the 6 MAGs and the 19 reference genomes. To further verify the quality of the MAG, we clustered the contigs using anvi’o’s *refine* module and sampled up to 200 proteins from each cluster. Each protein was compared against the UniRef90 database using DIAMOND (parameters: blastp -threads 10). Using the majority voted consensus lineage of up to three hits per protein (*e*-value threshold of 1e−20) with a majority threshold of 60 and a subsequent global majority vote using the same threshold, we assigned taxonomic lineages to each cluster.

### Analysis of TARA Ocean data

We assembled and analysed metagenomes from the TARA Ocean study PRJEB4352 using the same protocol as for the skin metagenomic data. We assembled and binned reads from ERR1700893, ERR1726523, ERR1726543, ERR1726560, ERR1726561, ERR1726573, ERR1726589, ERR1726593, ERR1726609 and ERR1726612. The study we selected has 912 runs associated, and we chose this subset of runs at random as we were limited by the large amount of memory and CPU time required for each assembly (for example, assembling ERR1726589 required 942Gb of RAM).

## Supplementary information

**Additional file 1.** Supplementary Figures.

**Additional file 2: Table S1.** Table of Genomes included in EukCC database. (CSV 17 kb)

**Additional file 3: Table S2.** Benchmark information, contamination from same clade. (CSV 1 kb)

**Additional file 4: Table S3.** Benchmark information, contamination from outside clade. (CSV 1 kb)

**Additional file 5: Table S4.** Cluster completeness contribution. (CSV 121 bytes)

**Additional file 6: Table S5.** Recovered MAGs metadata. (CSV 526 bytes)

**Additional file 7.** Review history

## Data Availability

The EukCC code is available through Github (https://github.com/Finn-Lab/EukCC) [50], and the version used in this article (version 0.1.5.1) is available under the DOI: 10.5281/zenodo.3886647 [51]. Documentation can be found at readthedocs (https://eukcc.readthedocs.io/en/latest). The EukCC database can be downloaded from http://ftp.ebi.ac.uk/pub/databases/metagenomics/eukcc/eukcc_db_v1.1.tar.gz. All MAGs have been submitted into ENA under the accession PRJEB38633 [52]. The TARA Ocean dataset used is available under the accession PRJEB4352.
