# Supplementary material for: Estimating the quality of eukaryotic genomes recovered from metagenomic analysis with EukCC
Source: Genome Biol. 2020 Sep 10;21:244. doi: 10.1186/s13059-020-02155-4 (PMC7488429; doi:10.1186/s13059-020-02155-4)
Supplement: Supplementary file 1 — Additional file 1. Supplementary Figures. [file 13059_2020_2155_MOESM1_ESM.pdf]

EukCC: Supplementary material

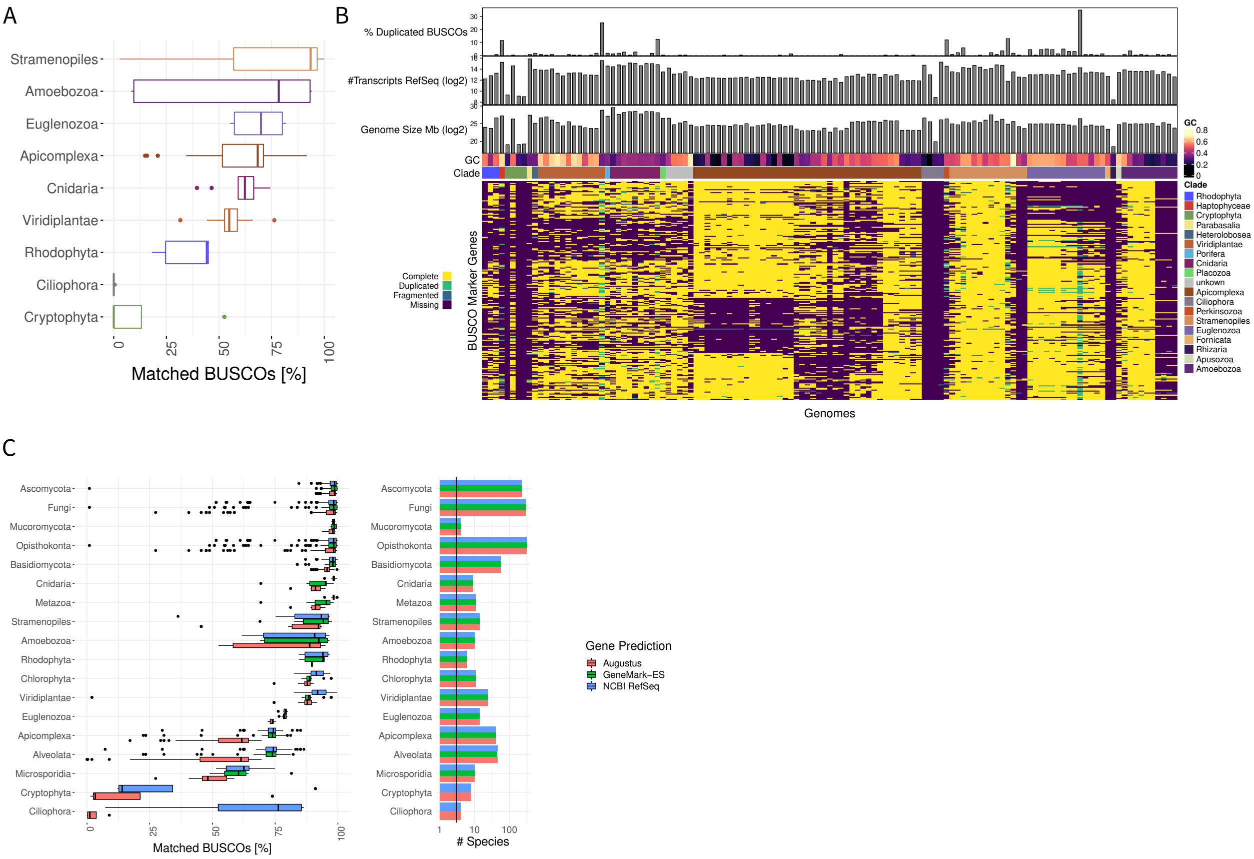

Fig. S1: **A**)Corresponding to the analysis in Figure **??** we used the BUSCO protist set to analyse a set of non Fungal genomes. In a number of protist clades, e.g. Apicomplexa and Amoebozoa, not all BUSCOs can be found. **B**) Matrix showing the breakdown of missing BUSCOs across different taxonomic groups. **C**) Running BUSCO using the 'eukaryota_odb9' set in *genome mode*, using GeneMark-ES, or using the NCBI RefSeq annotations, the number of found BUSCOs across these three gene callers is similar for Fungal clades, but increases when using GeneMark-ES or RefSeq for Euglenzoa and Apicomplexa.

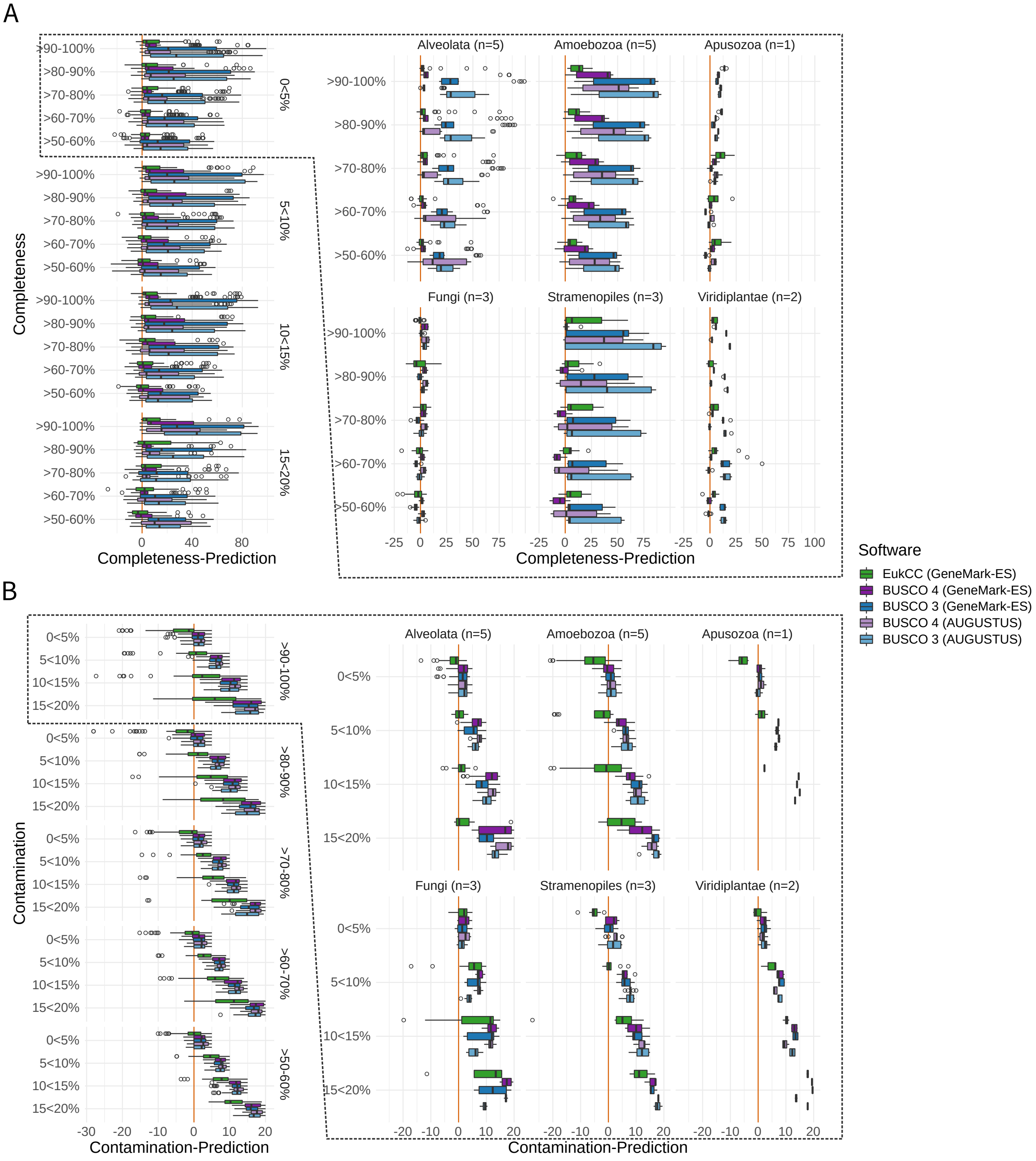

Fig. S2: A) For simulated genomes with a contamination below 5 % we split the panel 1 into the taxonomic clades (number of species per clade indicated by n). For the fungal clade both EukCC and BUSCO 3.1 and 4.0, independent on the gene caller, perform close to the expected value across a large range of simulated completeness. For Alveolates, Amoebozoa and Viridiplantae EukCC consistently performs better than BUSCO. Within the Stramenopiles EukCC both methods show a large variability in their performance. **B)** When looking at contamination estimated for highly complete genomes, BUSCO and EukCC perform best for low contamination ratios (<5 %). For Alveolata EukCC performs well across a large range of contamination. In the fungal clade both BUSCO and EukCC perform better for low contamination ratio and start to underestimate contamination with increasing amount of contamination. Within Amoebozoa and Viridiplantae EukCC, in contrast to BUSCO, tends to overestimate contamination by aprox 5 % for genomes with 0-5 % contamination.

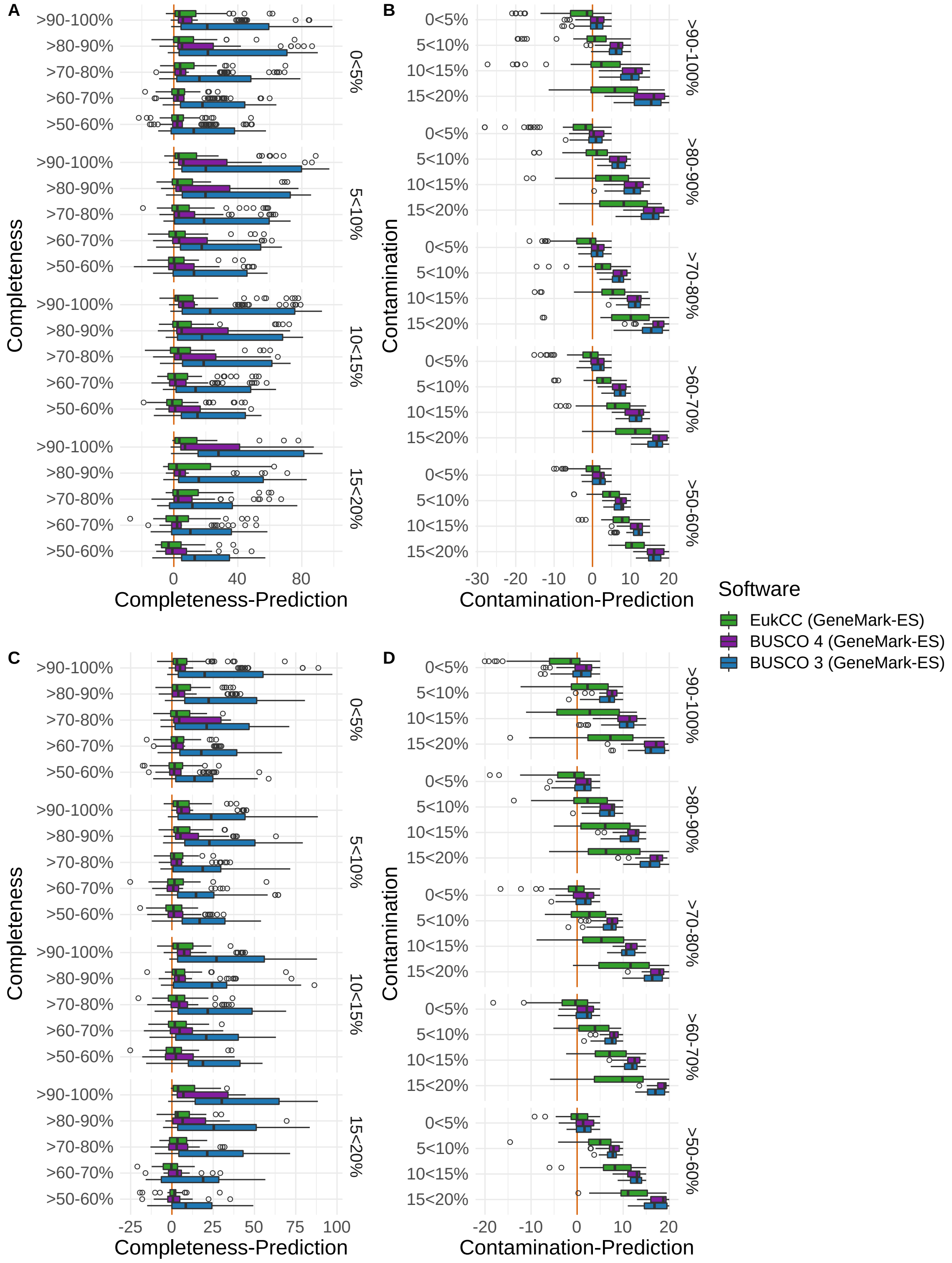

Fig. S3: We benchmarked EukCC and BUSCO 3.1 and 4.0 using genomes with added contamination from within the same clade, e.g. fungi contaminated with other fungal DNA (Panels A+B) as well as with contamination from outside the clade, e.g. Fungi contaminated with Viridiplantae (Panels C+D).

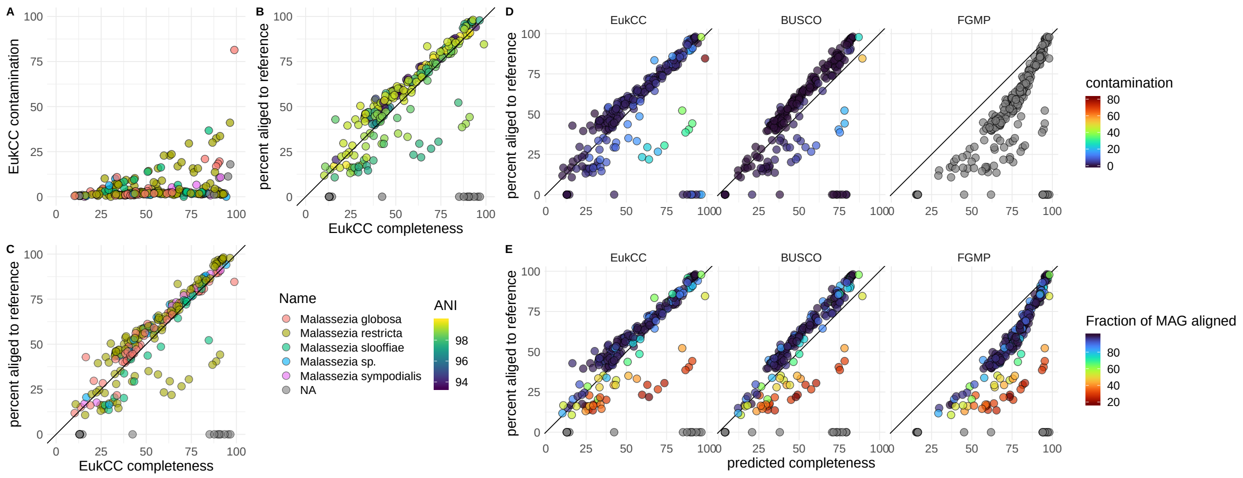

Fig. S4: A) Bins recovered from skin metagenomes were assigned to a reference genome and then estimated using EukCC in terms of completeness and contamination. Bins could be assigned to five different species of *Malassezia*. **B+C)** For bins that could be assigned to a reference we compared predicted completeness to how much of the reference could be aligned to the bin. For most bins EukCCs prediction is close to the aligned fragment. We see no signal when comparing the prediction of EukCC to the average nucleotide identity (ANI) between the MAG and the assigned reference genome neither when color coded by assigned species. **D)** We thus also checked completeness using BUSCO 4.0 and FGMP: BUSCO and EukCC performed comparable, both slightly underestimating completeness. FGMP overestimated completeness in almost all bins. Bins clearly overestimated by EukCC or BUSCO were also the most contaminated bins, which explains this behavior. **E)** When color coding bins by their percentage which could be aligned to the reference (Fraction of MAG aligned), well aligned bins are close to the diagonal and bins with a lower fraction of aligned DNA are commonly below the diagonal, which is in good agreement with the contamination estimate.

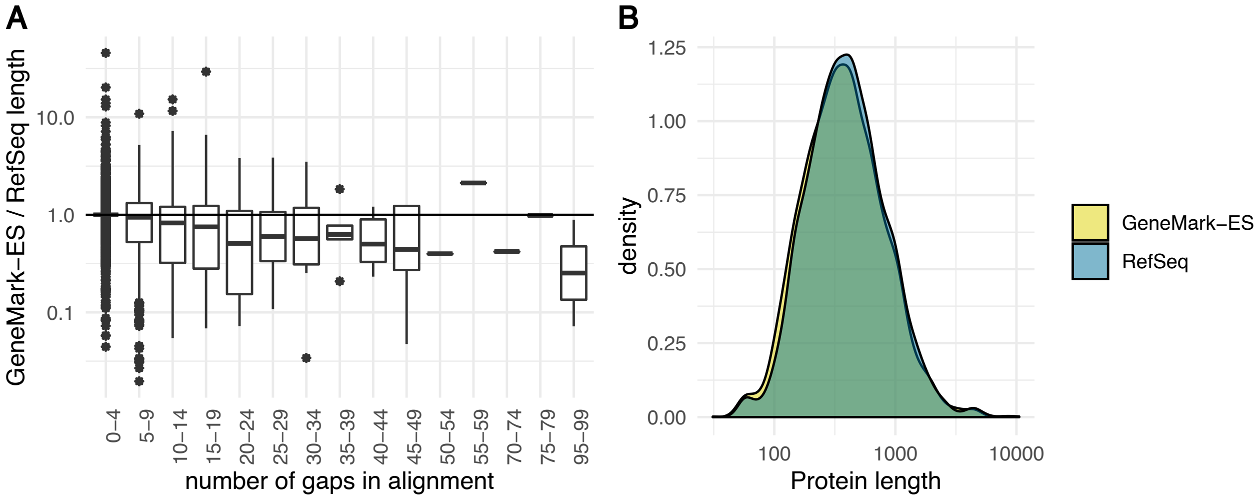

Fig. S5: Proteins predicted with GeneMark-ES that occured in a single copy in representative genomes across the eukaryotic genome (see Table S2 for used genomes) were blasted against their RefSeq proteome. For each protein the best hit (judged by e-value) with a maximal e-value cutoff of 1e-5 was chosen as the corresponding true protein. **A)** Each protein was aligned to its reference protein using MAFFT and gaps were counted. With increasing number of gaps the predicted proteins are shorter than their reference, suggesting that GeneMark-ES seems to miss some introns. **B)** We could not find a systematic bias between the protein length of UniProt or GeneMark-ES predicted proteins.

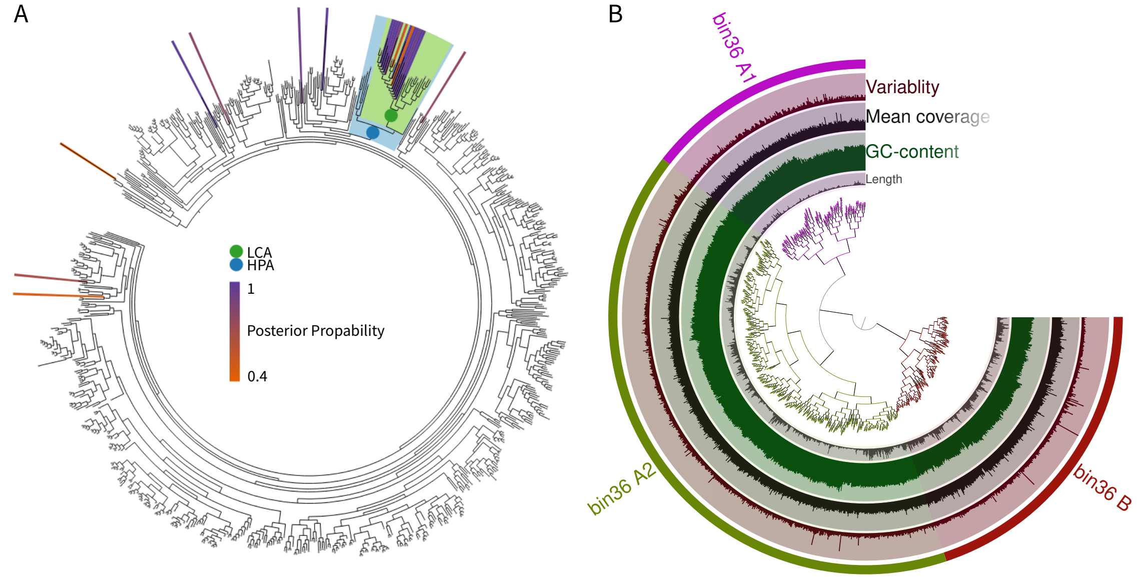

Fig. S6: **A)** Using EukCC to estimate the quality of the reported *Bathycoccus* MAG, pplacer placed the found marker proteins from the reference set mostly within a small range of the phylogenetic tree. Some outliers were ignored by EukCC while choosing the LCA set (green). **B)** The recovered *Bathycoccus* MAG from the TARA Ocean data was checked for quality issues using anvi'o. While the GC content and the coverage across all contigs is very uniform, anvi'o could form two large clusters. We taxonomically analysed both clusters and the indicated subclusters. All groups could be assigned the same taxonomic lineage, suggesting low amounts of contamination.

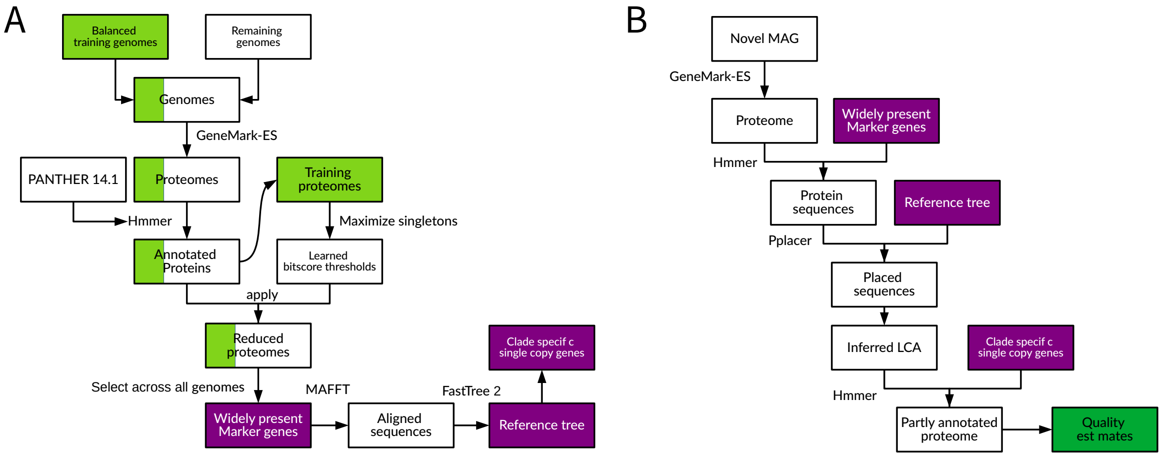

Fig. S7: **A)** EukCCs database is created by predicting proteomes using GeneMark-ES first. Proteomes are annotated using hmmer with PANTHER 14.1 families. A predefined subset of proteomes (green) are then used to learn bitscore thresholds for all profile hmms, maximizing the singleton prevalence across this set. Annotations are then filtered using the thresholds. By choosing widely present single copy genes to cover the entire genome space several times, we build a tree by first aligning proteins independently and then concatenating the alignments. **B)** EukCC searches for the widely defined marker genes to used pplacer to place a novel MAGs proteins into the reference tree. Choosing the lowest common ancestor set quality is computed.

Table S1: Genomes used to train the database. Genomes were excluded because of GeneMark-ES failure (labeled: gmes), because of long branches in the tree (tree) or because of problems during the set creation (set).

Table S2: Genomes used to evaluate EukCC. Each simulated MAG was based on a single RefSeq entry with added fragments from the contaminant. The specified BUSCO set was used to evaluate and the clade used to group results for Figure S2.

Table S3: Looking at the novel MAG in anvi'o, we saw several clusters (Figure 3). For each cluster we calculated the size and the contribution in completeness as well as the average marker density. Cluster A has the lowest contribution as well as the lowest marker density. Cluster C1, C2 and C2 are similar in density and comprise the largest percentage of the MAG. Cluster B is between A and C in all measures.
