# Supplementary material for: Estimating the quality of eukaryotic genomes recovered from metagenomic analysis with EukCC
Source: Genome Biol. 2020 Sep 10;21:244. doi: 10.1186/s13059-020-02155-4 (PMC7488429; doi:10.1186/s13059-020-02155-4)
Supplement: Supplementary file 7 — Additional file 7. Review history [file 13059_2020_2155_MOESM7_ESM.docx]

Review History

**First round of review**

**Reviewer 1**

**Were you able to assess all statistics in the manuscript, including the appropriateness of statistical tests used?** There are no statistics in the manuscript.

**Were you able to directly test the methods?** No.

**Comments to author:**

The manuscript entitled "Estimating the quality of eukaryotic genomes recovered from metagenomic analysis" by Paul Saary, Alex L. Mitchell and Robert D. Finn describes a bioinformatics tool, EukCC, that estimates completion and redundancy of eukaryotic genomes using at first a small set of widely occurring marker genes to determine the lineage with phylogenomics followed by a lineage-specific set of marker genes among a total of 477 gene markers selected by the authors. While the tool was built for metagenome-assembled genomes (MAGs) one can extract from metagenomes, it is fully compatible with genomes originating from other methods (e.g., single cell genomics, culture). Notably, while EukCC is highly similar to other tools already broadly used by the scientific community (e.g., BUSCO, CEGMA), the authors provided extensive benchmarking suggesting their tool is not only appropriate to study eukaryotic genomes of sufficient completion, but also more sensitive in many cases.

EukCC is a contribution to the slowly emerging field of genome-resolved metagenomics applied to eukaryotes, with well-described methods. It will be of use to those attempting to extract eukaryotic genomes from metagenomes, a small number of scientists for now but that will most likely grow in number in years to come. However, I have multiple concerns that need to be considered:

(1) While the software has the potential to be used by multiple bioinformatics labs moving forward, I failed to install it for testing. I asked multiple people to try and they failed as well. This is problematic.  

(2) Introduction is confusing. In my view, the flow of the introduction can be significantly improved for clarity. The manuscript is about estimating completion and contamination of eukaryotic genomes, with a focus on MAGs. Thus, the authors could start by describing widely used tools to estimate completion of eukaryotic genomes based on marker genes (especially BUSCO and CEGMA for eukaryotes, CheckM for Bacteria). The introduction could then focus on limits of current tools on the front of eukaryotes and the need for an improved methodology using latest genomic databases to define new lineage-specific gene marker sets: EukCC (same strategy as for CheckM). The introduction could then describe how genome-resolved metagenomics is now being used to characterize eukaryotic genomes without cultivation, and how EukCC can fit into this workflow, with case studies in the human skin and surface of the oceans as proofs of principle. I thank the authors for considering this flow.

(3) Completion limitations. It is unfortunate that EukCC does not work properly on genomes <50% in completion: "Across all simulated genomes, EukCC could estimate genome quality starting from a completeness of around 50 percent. Genomes less complete than this, were often not able to be processed using the self training mode of GeneMark-ES". First, this important point is clearly overlooked in the discussion. Second, a majority of eukaryotic MAGs extracted from metagenomic studies are going to be <50% complete. Thus, it is important the authors find a way to include MAGs of lower completion in their pipeline.

(4) MAG examples are very simple considering the complexity of the eukaryotic tree of life. On the second part of the manuscript dedicated to applicability of the method, it is unfortunate that fungi and Bathycoccus MAGs were used since they are very simple cases for which completion is highly accurate with BUSCO. This is because those lineages tend to have a majority of monoexonic genes, rendering gene calling very easy. Could the authors miss those MAGs using BUSCO? It seems quite unlikely. As a result, why using those examples to emphasize the utility of EukCC? In addition, the Bathycoccus MAG was most likely already characterized using genome-resolved metagenomics and TARA Oceans metagenomes but the authors failed to acknowledge this (https://www.nature.com/articles/s41564-018-0176-9).

(5) Underestimation of completion with EukCC. With the current strategy of EukCC, the contamination by distantly related eukaryotic MAGs is underestimated since the set of marker genes is lineage specific. In other words, contamination from distantly related eukaryotic genomes is more difficult to detect with this approach. In the legend of figure 2 (the section that relates to benchmarking contamination detection), authors write: "We fragmented the genomes and added varying amounts of contamination from another genome in the same clade". While very useful, missing is the same analysis but this time by adding contamination from other clades instead of the same clade. As the authors might understand, this is a critical analysis that needs to be incorporated for benchmarking. I think the authors for this extra-analysis and hope they see value in not overlooking this limitation of the approach on the front of contamination.

(6) More data is needed for TARA Oceans. At the end of the discussion, authors claim they are able with EukCC to screen large amount of eukaryotic MAGs from marine systems: "We have additionally demonstrated that current metagenomic techniques are also able to recover large fractions of eukaryotic genomes from more complex biomes, such as marine environment". However, authors only described a single MAG associated with a rather small, simple genome of Bathycoccus. Authors will need to share much more regarding TARA Oceans if they want to maintain this important claim. I kindly remind the authors here that ~70 eukaryotic MAGs have already been characterized from TARA Oceans in the past, including near-complete Bathycoccus MAGs and other ones, substantially longer.

\# Small points:

In figure 3C, what is the clustering used? Sequence composition or coverage? Why is there 5 selections since the authors describe 3 main clusters?

**Reviewer 2**

**Were you able to assess all statistics in the manuscript, including the appropriateness of statistical tests used?** No, do not feel adequately qualified to assess the statistics.

**Were you able to directly test the methods?** Yes.

**Comments to author:**

This is an excellent manuscript and tool, highly deserving of publication. It fills a needed niche and provides a robust, assessed approach.
I realize this preprint and manuscript was submitted several months ago, I am guilty of taking too long to review this and I apologize. My best attempt at making time for a review is as follows:

Species like Microsporidia are highly reduced so I am unsure if including them in the assessment is productive or at least should be more carefully noted, as Eukaryotes go they will always have a highly reduced recovered count even for conserved markers.

Worth mentioning GeneMark-ES fails on highly fragmented genomes and where there are not fragments > 10kb or so?
I would note that BUSCO4 does have a method now for automatic selection of appropriate marker set too. Not sure if that changes much in this manuscript approach but it is a noted deficiency made by the authors of the BUSCO approach. I don't know for sure how well the BUSCO4 approach compares to the pplacer/LCA approach applied here.

In my assessment the software is highly useful, the authors do a good job assessing their tool, applying it to real data, using simulated data via inserting random contamination. I think this is all around going to be highly useful for metagenome studies focusing on the eukaryotic fractio of metagenomes.

**Authors Response**

**Point-by-point responses to the reviewers’ comments:**

Responses to Reviewer 1:

(1) While the software has the potential to be used by multiple bioinformatics labs moving forward, I failed to install it for testing. I asked multiple people to try and they failed as well. This is problematic.

***Response: We were aware that EukCC can be a little tricky to install, based on the feedback from a number of users who did manage to install the software. We also know from a couple of usability bugs, that others have been successful in installing the software. To make the installation of EukCC easier, and address reviewer 1 issues, now have we submitted EukCC to bioconda and pypi, which will facilitate installation. We have updated our documentation accordingly.***

(2) Introduction is confusing. In my view, the flow of the introduction can be significantly improved for clarity. The manuscript is about estimating completion and contamination of eukaryotic genomes, with a focus on MAGs. Thus, the authors could start by describing widely used tools to estimate completion of eukaryotic genomes based on marker genes (especially BUSCO and CEGMA for eukaryotes, CheckM for Bacteria). The introduction could then focus on limits of current tools on the front of eukaryotes and the need for an improved methodology using latest genomic databases to define new lineage-specific gene marker sets: EukCC (same strategy as for CheckM). The introduction could then describe how genome-resolved metagenomics is now being used to characterize eukaryotic genomes without cultivation, and how EukCC can fit into this workflow, with case studies in the human skin and surface of the oceans as proofs of principle. I thank the authors for considering this flow.

***Response: Upon re-reading the introduction and in light of this reviewers comment, we have considerably reworked and streamlined the introduction.***

(3) Completion limitations. It is unfortunate that EukCC does not work properly on genomes <50% in completion: "Across all simulated genomes, EukCC could estimate genome quality starting from a completeness of around 50 percent. Genomes less complete than this, were often not able to be processed using the self training mode of GeneMark-ES". First, this important point is clearly overlooked in the discussion. Second, a majority of eukaryotic MAGs extracted from metagenomic studies are going to be <50% complete. Thus, it is important the authors find a way to include MAGs of lower completion in their pipeline.

***Response: EukCC’s limitation to estimate highly incomplete and contaminated bins is caused by the chosen gene predictor software, GeneMark-ES. In the latest version of EukCC, this limitation can be overcome by using EukCC with the ‘--pygmes’ flag: Using this mode gene predictions are aided by known models and thus work also for very incomplete bins. To clarify this point we expanded the discussion as suggested. This additional module is available when installing from either pip or via bioconda.***

(4) MAG examples are very simple considering the complexity of the eukaryotic tree of life. On the second part of the manuscript dedicated to applicability of the method, it is unfortunate that fungi and Bathycoccus MAGs were used since they are very simple cases for which completion is highly accurate with BUSCO. This is because those lineages tend to have a majority of monoexonic genes, rendering gene calling very easy. Could the authors miss those MAGs using BUSCO? It seems quite unlikely. As a result, why using those examples to emphasize the utility of EukCC? In addition, the Bathycoccus MAG was most likely already characterized using genome-resolved metagenomics and TARA Oceans metagenomes but the authors failed to acknowledge this (https://www.nature.com/articles/s41564-018-0176-9).

***Response: As pointed out the provided novel MAG examples are simple and, with certain adjustments to an automated workflow, could have been addressed using BUSCO. We want to highlight that this submission is a software article and not a survey of eukaryotic MAG’s. The chosen examples show the application of EukCC to be used in an automated workflow, screening a large number of candidate bins, without prior information, a feature BUSCO did not implement until BUSCO 4 until January 2020. Also, these examples were chosen such that the results of novel findings could be verified, and demonstrate the accuracy of EukCC when estimating genome quality. As suggested by reviewer 1 we included the collection of MAGs published by Delmont et al. 2018 in addition to the GenBank database, which corresponded to assemblies from the prokaryotic fractions from the Tara Oceans . Interestingly, theBathycoccus MAG described in this manuscript is similar to the MAG PSE_140, despite our assemblies focusing on a small subset of the eukaryotic fraction from Tara Oceans . We thus expanded the text to include a comparison between the two MAGs.***

(5) Underestimation of contamination with EukCC. With the current strategy of EukCC, the contamination by distantly related eukaryotic MAGs is underestimated since the set of marker genes is lineage specific. In other words, contamination from distantly related eukaryotic genomes is more difficult to detect with this approach. In the legend of figure 2 (the section that relates to benchmarking contamination detection), authors write: "We fragmented the genomes and added varying amounts of contamination from another genome in the same clade". While very useful, missing is the same analysis but this time by adding contamination from other clades instead of the same clade. As the authors might understand, this is a critical analysis that needs to be incorporated for benchmarking. I thank the authors for this extra-analysis and hope they see value in not overlooking this limitation of the approach on the front of contamination.

***Response: Estimating contamination from a foreign clade is a challenging problem, as marker genes from one clade do not have to be present in another. Also predicting proteins in hybrid genomes, might be confounded by the model learned by GeneMarkES. To verify that EukCC can handle such cases, we added a benchmark to our analysis that included more distantly related contaminating sequences, as suggested by the review. This additional benchmark highlighted that there were in fact only small changes in the tools ability to estimate completeness and contaminations between distant and close contaminating species.***

(6) More data is needed for TARA Oceans. At the end of the discussion, authors claim they are able with EukCC to screen large amount of eukaryotic MAGs from marine systems: "We have additionally demonstrated that current metagenomic techniques are also able to recover large fractions of eukaryotic genomes from more complex biomes, such as marine environment". However, authors only described a single MAG associated with a rather small, simple genome of Bathycoccus. Authors will need to share much more regarding TARA Oceans if they want to maintain this important claim. I kindly remind the authors here that ~70 eukaryotic MAGs have already been characterized from TARA Oceans in the past, including near-complete Bathycoccus MAGs and other ones, substantially longer.

***Response: The TARA Ocean project published over 900 datasets in the study we referenced. In this publication we looked at only 10 of these, primarily as this is a software paper. Recently, the study was fully assembled by Karin et al., 2020, which represents the biggest computational bottleneck, rather than running of EukCC. The Bathycoccus example is 3-4 times the size of bacterial genomes, so does represent a larger genome. We did not concluded that current techniques are able to exhaustively recover eukaryotic genomes, which in our experience will require advances in the assembly and the binning process of these diverse environments e.g. deeper sequencing, differential coverage binning and strategies such as co-assembly to yield MAGs of less abundant species. We have revised the wording of this statement as we were not meaning large numbers of Eukaryotes by the phrase “large fractions”, rather “near complete” eukaryotic genomes.***

Responses to Reviewer 2:

Species like Microsporidia are highly reduced so I am unsure if including them in the assessment is productive or at least should be more carefully noted, as Eukaryotes go they will always have a highly reduced recovered count even for conserved markers.

***Response: Microsporidia are one of the clades that we thought about a lot. We agree that they pose an extreme and special case within the single cellular eukaryotes. We chose to include them, as they might be part of a future metagenomic dataset. Any tool within this context will need to be able to handle bins from even the strangest of eukaryotes. Thus we feel including them in our analysis is wranted.***

Worth mentioning GeneMark-ES fails on highly fragmented genomes and where there are not fragments > 10kb or so?

***Response: Regarding the failure of GeneMark-ES on highly fragmented bins: As this is a point often coming up we worked on improving this step within the EukCC pipeline by implementing a new mode, which can in addition to the self training, use pre-trained models we obtained during development of EukCC. This mode can be enabled using `- -pygmes`. We updated the documentation and the paper accordingly.***

I would note that BUSCO4 does have a method now for automatic selection of appropriate marker set too. Not sure if that changes much in this manuscript approach but it is a noted deficiency made by the authors of the BUSCO approach. I don't know for sure how well the BUSCO4 approach compares to the pplacer/LCA approach applied here.

***Response: We were aware that BUSCO 4 was released while this manuscript was under review. We believe BUSCO 4 is a huge step in the right direction, improving the usability ofBUSCO a lot and to some extent closing the gap to EukCC in terms of accuracy, compared to BUSCO 3. Nevertheless, we felt it was important to be scientifically balanced and include BUSCO 4 in this manuscript. Furthermore, we are also aware that BUSCO 4 includes data that is in our benchmark, so the benchmark is not completely independent for BUSCO 4, where it is for EukCC. Nevertheless, while BUSCO 4 now performs much closer to EukCC, EukCC still outperforms BUSCO. The manuscript has been modified extensively to take into account BUSCO 4 in both the benchmarks and assessment of our MAGs.***

**Second round of review**

**Reviewer 1**

I appreciate all responses from the authors, as well as the improvements in term of writing and benchmarking. Most importantly, the software is now easy to install. I could test few eukaryotic MAGs with the tool, and the results are coherent with my expectations. It is also good that previously published Bathycoccus MAGs were included in the study (I note that TARA_01 is still considered “novel” in the discussion, despite a considerable overlap with a published one from TARA). I thank the authors for their work. I consider the manuscript has been substantially improved.

Small points:

Ln 81-82: please add references

Ln 121: please fix the writing.

As a last point that might go beyond the scope of this manuscript, yet is linked to it, I would like to emphasize that single copy core genes are often only found in a small subset of contigs in a given MAG (particularly true of the eukaryotes) and that, while tools such as EukCC or BUSCO 4 are very useful, a proper binning effort is still critical to minimize extent of real contamination (i.e., not only based on the single copy core genes). In that regard, differential coverage of contigs across as many relevant metagenomes as possible is a must. It is unfortunate such effort is not always being done for bacterial MAGs (the extent of "silent" contamination is large MAG databases is problematic and so far overlooked), and there is a chance now to get higher standards in the new and exciting front of eukaryotic genome-resolved metagenomics. This is particularly relevant to the last sentence of the discussion: "More importantly we showed that the combination of tools such as EukRep and EukCC can achieve comparable levels of genome quality compared with more manual approaches (as was performed for TARA_PSE_MAG_00140), by offering a scalable approach for the generation of eukaryotic genomes from metagenomic datasets". I personally do not fully share this point of view, however I understand the reasoning, especially regarding the scale.

**Authors Response**

**Point-by-point responses to the reviewers’ comments:**

Response to reviewer 1:

***We thank reviewer 1 for the continuous input of comments, which led to the improvement of our manuscript.***

"As a last point that might go beyond the scope of this manuscript, yet is linked to it, I would like to emphasize that single copy core genes are often only found in a small subset of contigs in a given MAG (particularly true of the eukaryotes) and that, while tools such as EukCC or BUSCO 4 are very useful, a proper binning effort is still critical to minimize extent of real contamination (i.e., not only based on the single copy core genes). In that regard, differential coverage of contigs across as many relevant metagenomes as possible is a must. It is unfortunate such effort is not always being done for bacterial MAGs (the extent of "silent" contamination is large MAG databases is problematic and so far overlooked), and there is a chance now to get higher standards in the new and exciting front of eukaryotic genome-resolved metagenomics. This is particularly relevant to the last sentence of the discussion: "More importantly we showed that the combination of tools such as EukRep and EukCC can achieve comparable levels of genome quality compared with more manual approaches (as was performed for TARA_PSE_MAG_00140), by offering a scalable approach for the generation of eukaryotic genomes from metagenomic datasets". I personally do not fully share this point of view, however I understand the reasoning, especially regarding the scale."

***Response: To address the issue of silent contamination EukCC starting in version 0.2 now reports the silent fraction of contigs as percentage DNA with no evaluated marker gene. In the previous version of the manuscript, we did evaluate the distribution of the marker genes across the genomes, and demonstrated that there was relatively even coverage across the genome. We have added a sentence to this manuscript highlighting this new feature in the output.***

***While we agree this and differential coverage could be possible avenues for further improvement of quality estimation tools such as EukCC, such metrics are sensitive to changes in the assembler as well as the binning approach and other parameters and thus a thorough evaluation of it is beyond the scope of this publication. There are currently over 300,000 metagenomic assemblies in ENA, so if one were to survey all of them for Eukaryotic genomes, this arguable requires automation, to at the very least select the best potential genomes. Manual curation may still uncover some errors, but we stand by our statement in the discussion.***

"I appreciate all responses from the authors, as well as the improvements in term of writing and benchmarking. Most importantly, the software is now easy to install. I could test few eukaryotic MAGs with the tool, and the results are coherent with my expectations. It is also good that previously published Bathycoccus MAGs were included in the study (I note that TARA_01 is still considered “novel” in the discussion, despite a considerable overlap with a published one from TARA). I thank the authors for their work. I consider the manuscript has been substantially improved."

***Response: We are pleased that this reviewer has now been able to cleanly install and test EukCC, and it meets their expectations. While the reported Bathycoccus MAG shares DNA with already reported MAGs the species boundaries for Eukaryotes are difficult to determine. We think the DNA of both MAGs are sufficiently different (10% unmatched) to call it ‘novel’ . We again would like to thank this reviewer again for their first round set of comments and are pleased that they feel the manuscript is ‘substantially improved’.***

"Small points: Ln 81-82: please add references

Ln 121: please fix the writing."

***Response: We have addressed both of these points in the revised manuscript.***
